# Towards Optimizing Sub-Normothermic Machine Perfusion in Fasciocutaneous Flaps: A Large Animal Study

**DOI:** 10.3390/bioengineering10121415

**Published:** 2023-12-12

**Authors:** Yanis Berkane, Alexandre G. Lellouch, Guillaume Goudot, Austin Shamlou, Irina Filz von Reiterdank, Marion Goutard, Pierre Tawa, Paul Girard, Nicolas Bertheuil, Basak E. Uygun, Mark A. Randolph, Jérôme Duisit, Curtis L. Cetrulo, Korkut Uygun

**Affiliations:** 1 Division of Plastic and Reconstructive Surgery, Vascularized Composite Allotransplantation Laboratory, Center for Transplantation Sciences, Massachusetts General Hospital, Boston, MA 02114, USA; alellouch@mgb.org (A.G.L.); ifilzvonreiterdank@mhg.harvard.edu (I.F.v.R.); marion.goutard0@gmail.com (M.G.); piotawa@gmail.com (P.T.); marandolph@mgh.harvard.edu (M.A.R.);; 2 Harvard Medical School, Boston, MA 02115, USA; basakuygun@mgh.harvard.edu; 3 Department of Plastic, Reconstructive, and Aesthetic Surgery, CHU de Rennes, Université de Rennes, 35000 Rennes, France; paul.girard@hotmail.fr (P.G.); nbertheuil@gmail.com (N.B.); jerome.duisit@gmail.com (J.D.); 4 Shriners Children’s Boston, Boston, MA 02114, USA; 5 SITI Laboratory, UMR1236, INSERM, Université de Rennes, 35000 Rennes, France; 6 Innovative Therapies in Haemostasis, INSERM UMR-S 1140, University of Paris, 75006 Paris, France; 7 Cardiology Division, Massachusetts General Hospital, Harvard Medical School, Boston, MA 02115, USA; ggoudot@mgh.harvard.edu; 8 INSERM U970 PARCC, Université Paris Cité, 75000 Paris, France; 9 Center for Engineering in Medicine and Surgery, Department of Surgery, Massachusetts General Hospital, Boston, MA 02115, USA; 10 University Medical Center Utrecht, 3584 Utrecht, The Netherlands; 11 Iris South Hospitals, 1040 Brussels, Belgium

**Keywords:** fasciocutaneous flaps, machine perfusion, ex vivo perfusion, vascularized composite allotransplantation, intermittent perfusion, machine perfusion, extracorporeal perfusion

## Abstract

Machine perfusion has developed rapidly since its first use in solid organ transplantation. Likewise, reconstructive surgery has kept pace, and ex vivo perfusion appears as a new trend in vascularized composite allotransplants preservation. In autologous reconstruction, fasciocutaneous flaps are now the gold standard due to their low morbidity (muscle sparing) and favorable functional and cosmetic results. However, failures still occasionally arise due to difficulties encountered with the vessels during free flap transfer. The development of machine perfusion procedures would make it possible to temporarily substitute or even avoid microsurgical anastomoses in certain complex cases. We performed oxygenated acellular sub-normothermic perfusions of fasciocutaneous flaps for 24 and 48 h in a porcine model and compared continuous and intermittent perfusion regimens. The monitored metrics included vascular resistance, edema, arteriovenous oxygen gas differentials, and metabolic parameters. A final histological assessment was performed. Porcine flaps which underwent successful oxygenated perfusion showed minimal or no signs of cell necrosis at the end of the perfusion. Intermittent perfusion allowed overall better results to be obtained at 24 h and extended perfusion duration. This work provides a strong foundation for further research and could lead to new and reliable reconstructive techniques.

## 1. Introduction

The advent of microsurgery was a turning point toward improving autologous reconstructions, allowing vascularized tissue transfers to occur at a distance from the donor site. Increasing anatomical knowledge and better microsurgery techniques have led to the fasciocutaneous flaps gradually replacing muscle-based reconstructions [1,2]. Additionally, the advent of perforator-based flaps [3,4,5,6] allowed for the reliability of these reconstructions to be increased while minimizing morbidity. Reliable perforator-based flaps used in clinical practice include the deep epigastric inferior perforator (DIEP) flap, which has surpassed rectus abdominis-based flaps for breast reconstruction [7], and antero-lateral tight or superficial circumflex iliac perforator flaps, which have become standard in limb reconstruction [8]. Nonetheless, reports suggest that 3 to 10% of free flaps still fail due to vascular complications [9,10,11,12,13,14,15]. Surgical revisions can be successful, but these attempts expose patients to delayed complications. Machine perfusion (MP) techniques could potentially increase the possibility of flap salvation after an initial failure of conventional microsurgery [16], for ex vivo thrombolysis [17,18], or even for fillet-flap preservation after major trauma leading to amputation [19,20,21]. In addition, some patients awaiting reconstruction are not eligible for free flap surgery because of their medical history. For instance, patients with extensive surgical and radiation histories of the cervical region (i.e., frozen necks) or diabetic patients with chronic wounds often present with unsuitable vascular networks [22,23].

One solution to circumvent these obstacles would be to master extracorporeal perfusion processes, thereby providing an exogenous supply of oxygen and nutrients to the flap and bridging the period necessary to reach flap autonomization/neo-vascularization and avoiding a vascularized transfer. Mastering a multi-day perfusion protocol [24] could be used for microsurgery-free flap reconstruction, as described by Wolff et al. [16,25,26]. Until now, they have been the first and only team to describe a clinical series of free fasciocutaneous flap reconstruction using exclusive extracorporeal perfusion. They were able to perform reconstructions of complex head, neck, and shoulder defects using free flaps with a fasciocutaneous component and no vascular anastomoses. Their innovative technique used anterolateral thigh, soleus, medial sural, radial forearm, and fibular vascularized fasciocutaneous paddles placed on the recipient site for 4 to 12 days until autonomization. However, their innovative approach will require important optimization to overcome the current limitations and lower the current complication rate of 67% observed in their series on partial flap loss [16,25,26]. On the other hand, Brouwers and Kruit have explored machine perfusion-based approaches in muscle flaps to study ex vivo thrombolysis in flap salvage [27], as well as for extended preservation as a relevant model for vascularized composite allotransplantation (VCA) [18,28]. Our team later explored subnormothermic machine perfusion techniques in rat limbs, providing a proof of concept of the use of machine perfusion in VCA, including bone components [29,30,31,32]. Overall, these techniques inspired by solid organ transplantation are developing as promising approaches in plastic and reconstructive surgery. However, experimental studies focusing on optimizing machine perfusion in fasciocutaneous flaps are still missing.

We performed this study using a porcine saphenous flap [33] to assess the possibility of using fasciocutaneous flap machine perfusion in a clinically relevant setting. We hypothesized that acellular sub-normothermic machine perfusion (SNMP) would suit the multiday perfusion of fasciocutaneous flaps. The objective was to optimize the ex vivo machine perfusion of fasciocutaneous flaps, describe the critical monitoring parameters, and compare the outcomes with continuous and intermittent perfusion. 

## 2. Materials and Methods

Twelve female 30–35 kg Yorkshire pigs were used for these experiments (12 flaps were included in the data). The authors followed the ARRIVE guidelines checklist [34]. Animals were housed with access to food and water according to the local Center for Comparative Medicine (CCM) conditions. After an acclimation period, the animals underwent unilateral procurement surgery under general anesthesia. The contralateral side was used by other research teams that were able to procure tissues and solid organs before euthanasia, in order to optimize the number of animals sacrificed within the research facility with authorization from the Institutional Animal Care and Use Committee (IACUC). Following all harvesting procedures, animals were euthanized according to the local veterinarian guidelines.

### 2.1. Flap Procurement Procedure

**Figure 1:**
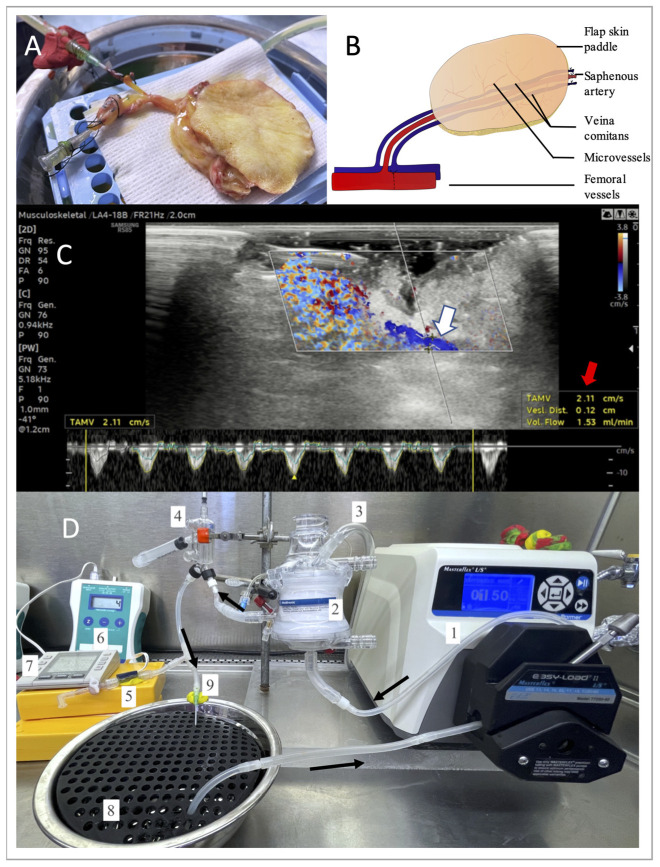
Surgical model and perfusion system. (**A**) Saphenous fasciocutaneous flap during the perfusion. (**B**) Schema of the flap vasculature. (**C**) Intra-operative ultrasound evaluation of the flow (color Doppler and pulsed Doppler) in the saphenous artery (white arrow), with an estimate of the flow rate (red arrow). (**D**) Perfusion system: **1:** peristaltic pump; **2**: oxygenator; **3:** oxygen flow; **4**: bubble trap; **5:** pressure sensor and inflow tap; **6:** pressure monitor; **7:** timer; **8**: perfusate reservoir; **9:** arterial cannula.

Unilateral axial saphenous fasciocutaneous flaps were harvested using our established model [33]. Vascular dissection was extended to the femoral vessels to allow for single cannulation of the two small veins in the flap (Figure 1A,B). After systemic IV administration of heparin (single dose of 100 UI/kg), the femoral vessels were dissected proximally and distally to the origin of the saphenous pedicle, ligated, and then divided. An 18 G catheter was inserted into the femoral vessels and secured with 3–0 silk ligatures. The flap was flushed through the artery with 30 to 50 mL of cold (4 °C) saline heparin (100 UI/mL) until clear venous return was achieved. Finally, the flap was weighed and transported to the perfusion system on ice. 

### 2.2. Machine Perfusion System

A customized machine perfusion system was designed using a roller pump (DRIVE MASTERFLEX L/S, Cole-Parmer, Vernon Hills, IL, USA), a hollow-fiber oxygenator (Affinity Pixie, Medtronic, Dublin, Ireland), inflow and outflow silicon tubing (Masterflex L/S 16, Cole-Parmer, Vernon Hills, IL, USA), a bubble trap (Radnoti 130149, Radnoti L.T.D, Dublin, Ireland), and a pressure transducer linked to a portable pressure monitor (PM-P-1, LSI, St Albans City, VT, USA). Filtration of potential debris was achieved by the hollow-fiber oxygenator, and no further filtration was performed during the perfusion. An oxygen tank (95% O_2_, 5% CO_2_) was connected to the dedicated valve on the oxygenator by silicon tubing, and the oxygen flow was set to 0.5 L/min. The pressure transducer was connected to the system upstream of the arterial cannula. A valve downstream of the pressure transducer was used to collect samples for the biochemical inflow measurements. The flap was suspended over a stainless-steel bowl filled with perfusate and laid on top of a perforated rack, allowing the outflow to collect in the solution reservoir freely (Figure 1D). A similar setup has been described for other models [31,32]. Samples of the outflow used in the biochemical analysis were procured from the venous cannula (18 G). The perfusion system (Figure 1D) was contained in a Class II biosafety cabinet. The tubing and the surgical bowl were autoclaved before each perfusion, and the flap was manipulated with sterile gloves and instruments. Perfusate solution was initially sterilized by filtration. Temperature was monitored using an external thermometer (Cole-Parmer, Traceable IR Thermometer) and was kept in the target range (19–21 °C) without intervention. Before the flap perfusion, the system pressure was measured at incremental flows from 0.5–4 mL/min using the same perfusate to correct the measurements with the system’s pressures.

### 2.3. Perfusate Solution

A custom-modified acellular Steen+ solution was used. Our team previously optimized this solution for vascularized composite allotransplant (VCA) preservation [28,31]. The main differences from the original Steen solution were the albumin concentration (increased to 15% in the Steen+, versus 7%) and the addition of 0.5% of 35 kDa polyethylene glycol [28] (Sigma-Aldrich, Saint-Louis, MO, USA). A Steen solution (7% bovine serum albumin), improved by adding broad-spectrum antibiotics (vancomycin 1 g/L and piperacillin–tazobactam 1 g/L), was used for the intermittent perfusion experiments. The perfusate was recirculated in a closed loop and exchanged every 24 h for the multi-day perfusion. Sodium bicarbonate (8.4%) was added to the solution to correct the pH. The optimal pH levels of the solution were between 7.1–7.4, varying with the CO_2_ levels.

### 2.4. Perfusion Monitoring

The following metrics were monitored throughout the perfusion period:

– Weight gain of the flap every 6 h;
– Perfusion parameters, including flow (mL/min) and measured and corrected pressures (mmHg);
– Resistances were calculated according to the formula R = P/Q (R: resistance (mmHg.min/mL), P: corrected pressure (mmHg), and Q: flow rate (mL/min));
– Biochemical parameters were repeatedly measured using a handheld analyzer (iStat 1, Abbott, Chicago, IL, USA). Inflow and outflow samples were collected for each time point and assessed the following measurements: pH, pO2 (mmHg), pCO_2_ (mmHg), lactate (mmol/L), [K+] (mmol/L), [Na+] (mmol/L), [HCO_3_^−^] (mmol/L), base excess (mmol/L), and glucose (mmol/L). Oxygen consumption was measured based on the difference in partial pressure between the inflow and outflow, the flow rate, and the initial weight using a modified Fick equation [35]. Similarly, glucose consumption was estimated as the inflow–outflow difference.

### 2.5. Determination of Flow Rates for Experiments

Two preliminary flap perfusions (not included in the data) were conducted for 12 h, allowing for the fine tuning of several perfusion parameters. The baseline flow rate of the saphenous artery was measured in vivo by ultrasound (Figure 1C), with artery identification by color Doppler and mean velocity quantification over time by pulsed Doppler. This optimization allowed for successful subsequent perfusions. 

### 2.6. Continuous Versus Intermittent Perfusion Protocols

Two non-pulsatile sub-normothermic perfusion regimens were compared. The first group received continuous perfusion (CP) with Steen+. The flow rate was manually adapted throughout the perfusion to keep the measured pressure between 30 and 55 mmHg based on our previous experience in other models [27,28]. A second group received intermittent perfusion (IP) with Steen. The perfusion rates chosen were based on a prior work of Wolff et al. [25], assuming a tolerance of the skin to ischemia. To address the ischemic complications observed in their series, the perfusion time/ischemic time ratio was increased to 30–45 min of perfusion followed by 75–90 min of ischemia. The perfusion parameters were assessed every 10 min during the perfusion phases, and the mean value per cycle was used for each time point.

For both groups, termination criteria were edema greater than 50% of the initial flap’s weight or inflow decreased to 50% of the initial value [17].

### 2.7. Statistical Analysis

All data were recorded in Excel (Microsoft, Redmond, WA, USA), and all statistical analyses were performed using Prism (v. 9.5.0, GraphPad Software, La Jolla, CA, USA). The alpha risk was fixed at 5%. For each variable measured during monitoring, the mean and standard error of the mean were determined. Linear regression was used to assess the stability over time in each group. Mann–Whitney U tests were performed to compare continuous quantitative variables between groups (non-paired, non-Gaussian, non-parametric rank distribution comparison).

## 3. Results

Six continuous and six intermittent sub-normothermic porcine flap perfusions were performed. The average surgical duration was 2.6 ± 0.5 h. The average skin paddle surface before the incision was 55.9 cm^2^ in the CP group and 64.5 cm^2^ in the IP group. The mean initial weight was 22.61 ± 3.98 g in the CP group and 30.95 ± 8.28 g in the IP group. All CP were stopped at t = 24 h due to reaching the termination criteria (weight at 24 h > 150% of the initial weight). The IP flaps were kept in the perfusion system for 24 to 72 h.

### 3.1. Perfusion Parameters

**Figure 2:**
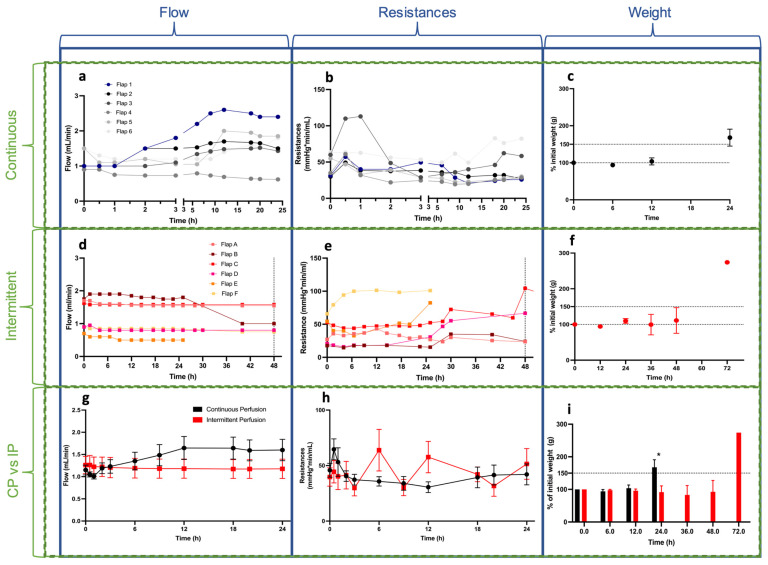
Perfusion parameters. The first, second, and third rows display the continuous perfusion group, the intermittent perfusion group, and the statistical analysis between groups, respectively. (**a**,**d**,**g**) Flow (**b**,**e**,**h**), resistance, and (**c**,**f**,**i**) weight variation are shown. Overall flow and resistances were comparable between groups. Edema was statistically lower in the I.P. group at 24 h. Mean weight gain at 36 and 48 h was lower in the I.P. group compared to the C.P. group at 24 h. Please note that the *x*-axis is split in (**a**,**b**) (continuous) into 0–3 h and 3–24 h to allow for better viewing of the initial three-hour results. For the I.P. group, the flow and resistance were the mean value per perfusion cycle (4 values per cycle). Values for the I.P. were collected for 48 h of perfusion. The third row shows mean values ± S.E.M. * statistically significant.

The perfusion parameter results are presented in Figure 2. The mean initial flow was 1.15 ± 0.27 mL/min in the CP group and 1.26 ± 0.48 mL/min in the IP group. The mean flow values at 24 h were 1.60 ± 0.59 and 1.14 ± 0.40 mL/min for the CP and IP groups, respectively. Comparison between groups showed no difference (*p*-values of 0.90 at t = 0 and 0.33 at t = 24 h). The mean initial vascular resistance was 45.78 ± 15.30 mmHg.min/mL in the CP group and 39.72 ± 20.33 in the IP group. The mean resistance at 24 h was 42.08 ± 23.17 and 51.48 ± 33.90 for the CP and IP groups, respectively. A trend showed an increase in resistance after 12 h, but the analyses at 0, 6, 12, and 24 h showed no significant differences between groups (*p* values of 0.39, 0.17, 0.07, and 0.70, respectively). In most flaps, high resistance was observed during the first 45 min before stabilization. Other perfusion models, such as the rat hindlimbs, showed evidence of similar patterns [31]. Interestingly, flaps perfused with the intermittent protocol showed lower vascular resistance during the perfusion cycles after repeated ischemia durations. Weight gain showed a nonsignificant increase after 12 h of perfusion (mean: 101.5% of the initial weight) in both groups, followed by a drastic rise at 24 h (164.1%) in the CP group, whereas the IP group showed very low edema at 24 h (109.92%, *p* = 0.04). At 48 h, the IP group showed interesting results, with mean flow, resistance, and weight values of 1.14 ± 0.40 mL/min, 54.72 ± 38.8 mmHg.min/mL, and 111.10 ± 80.66%, respectively.

### 3.2. Biochemical Parameters

**Figure 3:**
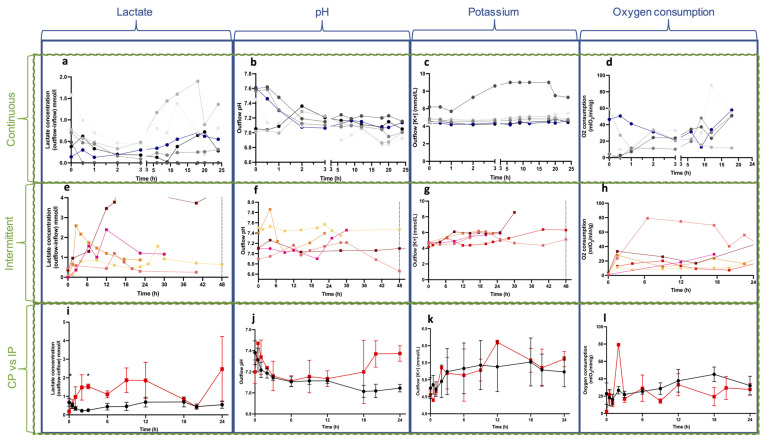
Biochemical analyses. The first, second, and third rows represent the continuous perfusion group, the intermittent perfusion group, and the statistical analysis between groups, respectively. (**a**,**e**,**i**) Lactate release showed higher values in the I.P. group, which was expected because of the ischemic periods. (**b**,**f**,**j**) pH levels were comparable between groups until t = 18 h, where the pH was higher in the I.P. group, which was linked to bicarbonate adjunction in one ischemic replicate. (**c**,**g**,**k**) Potassium levels were comparable between groups. (**d**,**h**,**l**) Oxygen consumption was measured with the following formula: O_2_cons = 100 × (Inflow O_2_-Outflow O_2_) × Flow rate × 0.0314/[initial weight of the flap] with O_2_cons in mlO_2_/min/g, InflowO_2_ and OutflowO_2_ in mmHg, flow rate in mL/min, and initial weight in grams. Please note that the x-axis is split in (**a**–**d**) (continuous) (0–3 h and 3–24 h), to allow for better viewing of the initial three-hour results. Values for the I.P. were collected for 48 h of perfusion. The third row shows mean values ± SEM.

Lactate was measured in the venous outflow, and the recirculating lactate measured in the inflow was subtracted. In the CP group, flaps #5 and #6 showed higher lactate values (up to 1.9 mmol/L), and these flaps were considered ischemic (Figure 3a). The drop in these two curves was due to the partial perfusate exchange. The mean initial lactate values were 0.60 ± 0.49 mmol/L in the CP and 0.18 ± 0.22 mmol/L in the IP group (Figure 3e). The mean values at 24 h were 0.55 ± 0.48 and 2.47 ± 3.93 mmol/L for the groups, respectively. A comparison between groups showed statistically significantly higher lactate values in the IP group, related to intermittent oxygenation (Figure 3i). The initial pH values varied between 7.1 and 7.4 depending on the pCO_2_ of the solution. Interestingly, the pH tended to stabilize over time to around 7.2 in both groups, except for the ischemic flaps (Figure 3b), due to metabolic acidosis. The potassium concentration (Figure 3c,g) measured in the outflow slightly increased during the first 24 h of perfusion, but not outside of average physiological values (3.5–5 mmol/L), apart from one flap (#1) in the CP group, which was determined to be contaminated with cleaning solution residues. The mean initial potassium concentration was 4.75 ± 0.75 mmol/L in the CP group and 4.56 ± 0.24 mmol/L in the IP group. The mean potassium concentrations at 24 h were 5.23 ± 1.07 mmol/L and 5.61 ± 0.50 mmol/L in the CP and IP groups, respectively. No statistical difference was found between groups during the 24 h period (Figure 3k). Figure 3d shows an increased O_2_ consumption beyond 12 h of perfusion in the CP group, but no statistical difference was found between the groups (Figure 3l). Oxygen consumption dropped after 8 h in flap #4 (CP group) due to bacterial growth in the bubble trap. Glucose consumption typically decreased during the first hour of perfusion before stabilizing at low values. The glucose consumption in flap #4 (CP group) reached high values after 7 h, and this was associated with bacterial infection.

## 4. Discussion

**Figure 4:**
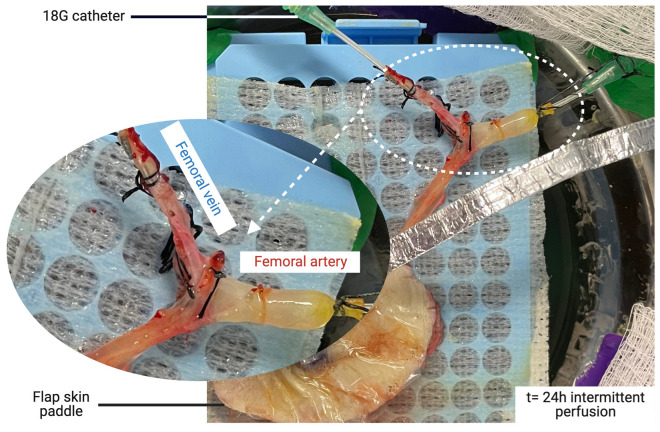
Macroscopic aspect of the cannulated femoral vessels following 24 h of intermittent perfusion. The vein was cannulated to facilitate the procurement of the outflow sample from both veina comitans. Note that the positioning of the femoral vessels was adjusted while monitoring the system’s pressure to allow for perfusion with minimal mechanical resistance.

In this work, we presented the setup and the main parameters for the ex vivo perfusion of fasciocutaneous flaps. Continuous perfusion was performed for 24 h in the first group (CP). Intermittent perfusion (IP) was studied in the second group until perfusion failure was reached. This study design allowed for a comparison of the two different groups in the first 24 h (Figure 4). A key goal was to show that acellular perfusion can be used for perfusing this type of flap. Preserving fasciocutaneous flaps for short durations (12–24 h) could be used for immediate clinical applications, such as complex free flap surgeries or revision surgeries, to decrease the ischemic time during preparation of the recipient site [36]. Ex vivo perfusion could also be used to preserve fillet flaps procured on amputated limbs following major traumas as an example of a tissue-sparing procedure [19,20,21]. Another application is ex vivo thrombolysis in compromised flaps, as previously described in a swine musculocutaneous flap model [17]. Our work can also inform future ex vivo perfusion studies, which is a current trend in the field of reconstructive surgery [37].

**Figure A1:**
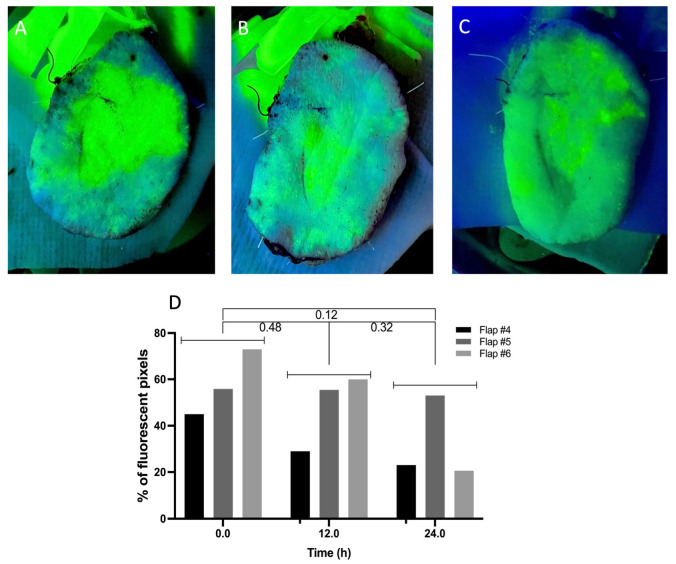
Flap fluorescein angiography. Aspects of the flap angiographies at (**A**) t = 0; (**B**) t = 12 h and (**C**) t = 24 h. (**D**) A non-paired *t*-test showed no statistically significant difference between the 3 time points. Mean initial angiographies in the C.P. group (t = 0) showed 55.80 ± 14.39% green fluorescent pixels on the whole flap skin paddle, confirming that the arterial flow reached the skin paddle. Mean values at 12 h and 24 h were 48.30 ± 16.90% and 32.20 ± 18.10%, respectively.

**Figure A2:**
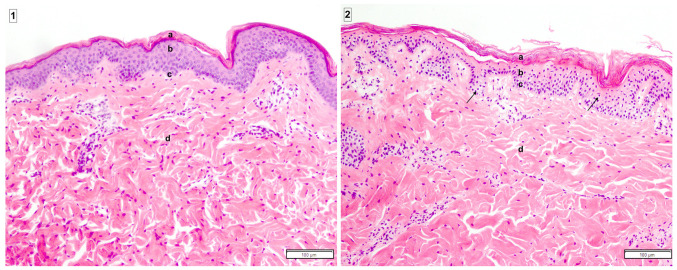
Tissue samples and histology. Legend: H&E staining of the fasciocutaneous flap skin after 24 h of continuous perfusion (**1**) and after 24 h of static storage at 21 °C (**2**). This photograph of H&E-stained tissue reveals the epidermis and partial dermis. The different cellular components can be visualized: epidermal stratum corneum (a), epidermal stratum spinosum (b), epidermal stratum basale (c), and dermis containing fibroblasts (d). The stratum spinosum and stratum basale in the control group (**2**) show minor apoptosis (black arrow). No necrosis is shown in any of the groups.

To assess the micro-vascularization of the skin paddle, fluorescein angiography was performed (Figure A1, Appendix A). It is interesting to note that the initial fluorescent surface was not 100%. The model itself could explain this: the microvessels dedicated to the flap are known to be highly dynamic, as described by Saint-Cyr and Rohrich with the perforasome theory [38]. Machine perfusion leads to increased resistance in other models [31,39], and this could explain the limited area reached by the fluorescence. It is also likely that the fluorescein angiography itself may cause this result, as it has been reported that fluorescein only assesses the deep dermal plexus [40]. It would be interesting to improve this microvasculature assessment by performing indocyanine green angiography (ICG) [40]. Histology showed no difference between any of the flaps in either perfused group (Figure A2, Appendix A), revealing edema, but no signs of apoptosis.

These preliminary experiments showed us the importance of the initial flow value on resistance in the flaps, reflective of the microvasculature. Since each flap has its own specific vascular compliance and anatomy, we recommend an intra-operative ultrasound examination for each new flap model to confirm its quality and estimate the initial arterial rate [41]. Biochemical measurements indicated potential ischemia, even in flaps with no muscle. Elevated lactate may also suggest a bacterial infection consuming oxygen, as seen in two preliminary flap perfusions where antibiotics corrected a drop in inflow oxygen (data not included). Untreated, prolonged low oxygenation of the inflow could result in lactic acidosis due to anaerobic metabolism [42]. This last point shows that critical care must be taken to prevent the perfusate from becoming contaminated. Therefore, for clinical applications, the perfusate should be micro-filtrated and discarded, and should not be recirculated. We used biochemical metrics such as potassium, lactate, pH, and oxygen consumption by translation from other VCA models [31,32,43]. These metabolic outcomes have been shown to be relevant in solid organ preservation [35,44], but other parameters may be suitable for fasciocutaneous flaps. Changes in ATP levels have been described in several models [45,46] and could be of interest to improve perfused flap monitoring, but this seems to be difficult to implement in clinical settings. All parameters described in our methods can be monitored using handheld and light devices, making them relevant for bedside applications.

Meyers et al., have recently shown that weight gain is an early marker of perfusion failure [47]. The overall analysis of our presented data suggests that both continuous and intermittent oxygenated acellular perfusion can be successful for short durations of less than 12 h, and that intermittent perfusion seems better for longer durations, potentially because it preserves the vascular tree, allowing for lower vascular resistance and edema. A correlation between these two parameters has been described previously by Dr. Pomahac’s team in a pig hindlimb model [48]. Our findings confirmed their results by showing a gradual parallel increase in weight gain and vascular resistance. In order to reach several days of optimized perfusion, further studies should therefore focus on better protecting the microvasculature to prevent interstitial edema and increased resistance.

We chose to compare continuous and intermittent perfusion regimens for several reasons: Current machine perfusion techniques in solid organs, but also in vascularized composite allografts, all use continuous perfusion to constantly provide oxygen and nutrients while constantly clearing toxic metabolites. On the other hand, intermittent perfusion in the specific case of fasciocutaneous flaps is interesting to explore: (i) the absence of muscle makes the ischemic phases acceptable; (ii) the intermittent perfusion allows for ischemic preconditioning on the flap, which can expedite the neo-vascularization process and ensure autonomization at the end of the machine perfusion period; and (iii) the logistics at the patient’s bedside would gain convenience, since intermittent perfusion would allow for mobilization and walking during the OFF phases, helping to decrease decubitus complications. Therefore, it seemed critical to compare both perfusion settings.

To our knowledge, this work is the first description of ex vivo perfusion of fasciocutaneous flaps in a large animal model. Muscle-sparing flaps seem to be the most clinically relevant to modern reconstructive techniques in plastic surgery [1,2,49]. Kruit et al. [28,50] first demonstrated perfusion success in porcine musculocutaneous flaps, allowing for 18 h of preservation before replantation. They compared two different commercialized perfusates, but their work did not focus on the perfusion parameters. Moreover, musculocutaneous flaps differ from pure fasciocutaneous flaps due to the presence of multiple perforator vessels that provide adequate vascularization to the skin paddle, but with a lower tolerance of the muscle to ischemia. Performing machine perfusion of fasciocutaneous flaps appears to be safe for reconstructive surgery applications, and this was the focus of our study because of the potential for immediate implementation in plastic surgery [2,51,52]. Ozturk et al., described the perfusion of five freshly harvested DIEP flaps on patients undergoing abdominoplasty [53]. They used fresh whole blood and were able to keep the flaps perfused for 4 to 5 days. However, they did not address the ideal flow rate or pressure parameters, which are critical for reproducibility. Additionally, the use of whole blood could be limiting, both in terms of safety and logistics for clinical use. We expect that acellular perfusion would be preferable for fasciocutaneous flap perfusion, limiting the cost and risk of infectious disease transmission, as shown by Wolff et al., in vivo [25]. To address this last point, it seems necessary to compare different perfusate solutions, including testing of potential artificial oxygen carriers.

This preliminary study has several limitations that should be addressed in the future. Firstly, a larger cohort would have provided better power for statistical analysis. Additionally, the contributions of angiography (Figure A2, Appendix A) were minor and limited to confirming arterial flow. Using ICG for such ex vivo flap perfusions could permit better monitoring of skin perfusion. Another point is the temperature, which was set as sub-normothermic (19–21 °C) and could have influenced the flap’s micro-vascularization [54]. Normothermic perfusion could improve the skin paddle’s vascularization. However, choosing a sub-normothermic perfusion permits using an acellular perfusate solution because of the lower metabolism [55,56], avoiding safety-related concerns regarding blood products. Moreover, the bacterial hazard needs to be addressed carefully. We modified our protocol by using piperacillin–tazobactam and vancomycin in our perfusate based on preliminary cases in which likely bacterial infections were observed. Another limitation is the absence of microscopic assessment of endothelial injuries following perfusion, which could potentially explain the edema and the perfusion duration limitation. Adding a sequence of normothermic blood reperfusion at the end of the preservation period could unveil ischemia–reperfusion injuries and increase the significance of this work, and should be explored in subsequent studies. To date, only a few publications have focused on endothelial cells during MP [57,58]. Finally, comparing extracorporeal perfusion protocols with conventional microsurgery could be interesting (outcomes, safety, cost-effectiveness…), but it seems that this innovation should be, at least initially, exclusively restricted to patients disqualified for microsurgical free flaps or for free flap salvage attempts (thrombolysis). Therefore, no comparison should be performed yet between these approaches during the optimization process.

This study was inspired by pioneering works by Wolff et al., who described the cases of six patients who benefited from extracorporeal perfusion techniques for reconstruction of the neck with fasciocutaneous flaps [16,25,26]. The patients in their series eventually healed, but four of six experienced partial or subtotal flap loss. This study did not evaluate certain parameters, such as perfusion rhythm, frequency, solute type, and total perfusion duration, which could have added benefits to avoid partial ischemic complications. Our objective was to optimize perfusion in a clinically relevant model. We found that intermittent perfusion seemed more suitable than continuous perfusion for multi-day perfusion based on vascular resistance and edema monitoring.

To further optimize the promising approach of intermittent flap perfusion, it is crucial to investigate the impact of perfusion/ischemia rates on flap perfusion quality. Several preclinical models already exist [59,60,61], and the new perspectives on reconstruction should push researchers to delve into this matter. Further research should also explore the healing capacity of flaps following extended perfusion preservation, as well as the endothelial injuries provoked by machine perfusion shear stress, which could explain the current limitation in perfusion duration due to weight gain by extravascular perfusate leakage. This study acts as a strong foundation for more studies, which will be needed in order to provide a reliable protocol allowing fasciocutaneous flap perfusions for extended durations, therefore enabling microsurgery-free reconstruction without ischemic complications.

## 5. Conclusions

Fasciocutaneous flaps can be preserved using continuous acellular subnormothermic machine perfusion for 12 h. Intermittent perfusion permitted up to 48 h of flap preservation. This strategy can allow for flap salvage using ex vivo thrombolysis, or even flap preservation before replantation in complex cases. Further research should aim for longer perfusion durations, eventually leading to optimizing anastomoses-free flap transfer reconstructions.

## 6. Patents

The authors declare U.S. Patent Application No. 63/377,519, filed 28 September 2023, as relevant to the work included in this manuscript.

## Data Availability

Data can be provided by the corresponding authors on demand.

## Appendix A

(1) **Perfusion quality assessment through vascular angiography.**

**Methods**: To monitor the flap microvasculature, angiographies were performed by manually injecting 0.3 mL of 5% fluorescein through the arterial cannula and exposing the flap to a Wood UV light (365 nm peak wavelength). High-resolution photographs of the skin paddle fluorescence were then taken at t = 0, t = 12 h, and t = 24 h. Pixel-analyzing software (v. 2.10., GNU Image Manipulation Program, Berkeley, CA, USA) was used to measure the percentage of fluorescent pixels inside the flap at each time point. A pre-injection photograph was taken before each angiograph in order to exclude residual fluorescence from the previous time point. The outflow was discarded for 30 s after the angiography to minimize fluorescein recirculation. Flap angiography was performed in the continuous perfusion group and was not repeated in the intermittent group.

(2) **Tissue samples and Histology:**

**Methods**: Full-thickness punch biopsies were collected at t = 0 (initial skin controls) and t = end of perfusion (24, 48, or 72 h) on the perfused flaps. Biopsies were fixed in 10% formalin, embedded in paraffin, and stained with hematoxylin and eosin (H&E) for basic pathology assessment. Further control flaps consisted of native skin (t = 0), static cold-stored flaps (4 °C, in Custodiol, Essential Pharmaceuticals LLC, Durham, NC, USA), and non-perfused flaps kept at room temperature (19–21 °C) for 24 h. The slides were analyzed by an experimented pathologist. A pathologic component scoring system was used to compare the samples [62]. Apoptotic cells per field (C.P.F) were counted and showed negative results in all flaps of both experimental groups at the end of the perfusion (24 h (Figure A2(1)), 48 or 72 h), in static cold-stored flaps at 4 °C (*n* = 3), and in native control flaps (*n* = 3). Only the non-perfused control flaps (*n* = 3) that were static and stored at room temperature (21 °C) showed minor apoptosis (Figure A2(2)). The component pathology score showed no difference between any of the groups. This limited contribution of histology may be due to the absence of normothermic blood reperfusion, which will be incorporated into our future experiments.
